# Prevalence of Risk for Obstructive Sleep Apnea Syndrome and Association With Risk Factors in Primary Care

**DOI:** 10.5935/abc.20160061

**Published:** 2016-06

**Authors:** Kenia Vieira da Silva, Maria Luiza Garcia Rosa, Antônio José Lagoeiro Jorge, Adson Renato Leite, Dayse Mary Silva Correia, Davi de Sá Silva, Diego Bragatto Cetto, Andreia da Paz Brum, Pedro Silveira Netto, Gustavo Domingos Rodrigues

**Affiliations:** 1 Departamento de Epidemiologia e Bioestatística - Universidade Federal Fluminense, Niterói, RJ - Brazil; 2 Departamento de Medicina Clínica - Universidade Federal Fluminense, Niterói, RJ - Brazil; 3 Departamento de Fundamentos de Enfermagem e Administração - Universidade Federal Fluminense, Niterói, RJ - Brazil

**Keywords:** Sleep Apnea Obstructive, Risk Factors, Prevalence, Surveys and Questionnaires

## Abstract

**Background:**

Obstructive sleep apnea syndrome (OSAS) is a chronic, progressive disease with high morbidity and mortality. It is underdiagnosed, especially among women.

**Objective:**

To study the prevalence of high risk for OSAS globally and for the Berlin Questionnaire (BQ) categories, and to evaluate the reliability of the BQ use in the population studied.

**Methods:**

Observational, cross-sectional study with individuals from the Niterói Family Doctor Program, randomly selected, aged between 45 and 99 years. The visits occurred between August/2011 and December/2012. Variables associated with each BQ category and with high risk for OSAS (global) were included in logistic regression models (p < 0.05).

**Results:**

Of the total (616), 403 individuals (65.4%) reported snoring. The prevalence of high risk for OSA was 42.4%, being 49.7% for category I, 10.2% for category II and 77.6% for category III.

**Conclusion:**

BQ showed an acceptable reliability after excluding the questions Has anyone noticed that you stop breathing during your sleep? and Have you ever dozed off or fallen asleep while driving?. This should be tested in further studies with samples mostly comprised of women and low educational level individuals. Given the burden of OSAS-related diseases and risks, studies should be conducted to validate new tools and to adapt BQ to better screen OSAS.

## Introduction

Obstructive sleep apnea syndrome (OSAS) is a chronic and progressive disease of increasing importance, because of its neurocognitive and cardiovascular sequelae, such as systemic arterial hypertension (SAH).^1^ It is underdiagnosed, mainly among women.^2^

Obstructive sleep apnea syndrome is characterized by repeated episodes of complete or partial airflow cessation in the upper airways (apnea and hypopnea, respectively). Such changes are due to complete or partial airflow obstruction at the pharynx level, often resulting in oxygen desaturation and brief awakenings from sleep (arousals).^2^

In addition to polysomnography, considered gold standard for the diagnosis of OSAS, some tools, such as scales, despite not diagnosing the disorder, indicate the risk for OSAS. Berlin Questionnaire (BQ) is one of them. It comprises three categories of questions, which include snoring, daytime sleepiness and diagnosis of hypertension and obesity.^3^

In Brazil, we identified only one study estimating the prevalence of high risk for OSAS in the general population, conducted in the city of São Paulo.^4^ Considering the high prevalence of hypertension and the need to better understand the behavior of the BQ in our population, a more careful investigation is certainly extremely useful.

The present study was aimed at estimating the prevalence of high risk for OSAS per BQ category and globally, in addition to assessing the reliability of BQ in a population cared for by the Niterói Family Doctor Program (FDP), Rio de Janeiro state, Brazil.

## Methods

The present study is part of the DIGITALIS Trial,^5^ a cross-sectional study of a random population sample registered in the Niterói FDP, including individuals of both sexes, aged from 45 to 99 years. Medical and nurse visits were appointed at selected FDP healthcare units from August 2011 to November 2012, where blood and urine samples were collected, electrocardiography and echocardiography performed, and a questionnaire specifically elaborated for the study with validated tools, such as the BQ, was applied. The researchers were trained in the procedures elaborated and tested in the pilot-study, carried out in a FDP healthcare unit not included in this study.

Initially, 942 individuals were invited, and 616 attended the appointments, completed the questionnaire, underwent anthropometric and clinical examinations, being included in this study (35% missed the appointment). Table 1 shows sex and age differences of those attending and completing the investigation and those who did not.

**Table 1:** Characteristics of the individuals invited to participate in the study (assessed and not assessed)

	**Participants with complete information**	**Individuals who refused to participate or those with incomplete information**
	**N (%)**	**N (%)**
**Sex**		
Female	381 (61.9)	174 (53.4)
Male	235 (38.1)	152 (46.6)
**Age group**		
45-49 years	110 (17.9)	70 (23.0)
50-59 years	246 (39.9)	105 (34.5)
60-69 years	150 (24.4)	65 (21.4)
70-79 years	86 (14.0)	47 (15.5)
80-99 years	24 (3.9)	17 (5.6)
Diabetes	151 (24.8)	
Hypertension	448 (72.7)	
Obesity (BMI≥30)	189 (30.7)	

BMI: body mass index.

**Endpoint:** High risk for OSAS measured via BQ. The BQ comprises 10 items, organized into three categories concerning snoring and apnea (5 items), daytime sleepiness (4 items) with a subquestion about sleepiness while driving (nodding off while driving a motor vehicle) and history of SAH or obesity (1 item). Risk classification (high risk *versus* non high risk) was based on the responses in each category, as follows: category I - persistent symptoms (>3-4 times/week) in at least 2 questions; category II - persistent symptoms (>3-4 times/week) with report of excessive daytime sleepiness or sleepiness while driving a motor vehicle, or both; category III - history of SAH or body mass index (BMI) ≥30 kg/m^2^. Individuals at high risk for OSAS were those with positive scoring in at least two BQ categories.^4,6^

**Exposure**. Age: was recorded in complete years at the time of the appointment and categorized into 10-year age ranges. Type 2 diabetes mellitus (DM): report of previous medical diagnosis of DM, fasting blood glucose ≥ 126 mg/dL measured at the time of the appointment, or use of antidiabetic medications. Arterial hypertension: previous diagnosis of SAH, systolic blood pressure (SBP) ≥ 140 mm Hg and/or diastolic blood pressure (DBP) ≥ 90 mm Hg measured at the time of the appointment, or regular use of anti-hypertensive drugs. Body mass index ≥30 kg/m^2^ was used to define obesity.

### Statistical analysis

We calculated the absolute and relative frequencies of the participants' characteristics, of the responses considered positive according to the BQ score, of the risk categories and of high risk for OSAS (global). Differences were tested as follows: between the proportions, by using Pearson chi-square test, with continuity correction for dichotomous risk variables; and between the means, by using non-paired Student *t* test. Variables associated with each category and conveying high risk for OSAS (global), with 0.20 significance in the difference between proportions or means, were included in logistic regression models, when statistical significance was established as <0.05. Because the presence of SAH or obesity (BMI) defines category III, those two variables were not assessed on raw and adjusted analysis of category III. All analyses were performed with the SPSS program, version 21 (IBM Corp. Released 2012. IBM SPSS Statistics for Windows, Version 21.0. Armonk, NY: IBM Corp).

### Ethical considerations

This study was conducted according to the principles established in CONEP Resolution 466/2012.

This study protocol was submitted to the Research Ethics Committee of the Medical School of the Antônio Pedro University-affiliated Hospital, and approved (CAAE:0077.0.258.000-10).

## Results

The sample of 616 individuals included in this study had the following characteristics: female sex, 61.9%; mean age, 59.1±10.20 years; elementary educational level, 68%; hypertensive, 72.7%; obese, 30.7%; and diabetic, 24.8%. Individuals of the two extreme age groups assessed comprised most of those excluded from the analysis.

Table 2 shows the scores of BQ responses and the prevalence of high risk for OSAS per BQ category and globally. Of all individuals assessed, 403 individuals (65.4%) reported snoring. Three of four responses of category I scored between 22% and 38.5%, while for the question *Has anyone noticed that you stop breathing during your sleep?*, only 1.8% of the responses scored. In category II, two of the three questions scored approximately 12%, but only 3.4% scored the question *Have you ever nodded off or fallen asleep while driving a vehicle?*. The global prevalence of high risk for OSAS was 42.4%, with 49.7% prevalence in category I, 10.2% in category II, and 77.6% in category III. The prevalence of high risk for OSAS in category I, according to age groups, had a bell shape curve (p<0.01) and was higher among obese individuals (p<0.01). Only sex associated with high risk for OSAS in category II (p<0.1 and >0.05). The characteristics 'female sex', 'advanced age' and 'DM' showed statistically significant association with high risk for OSAS in category III (hypertension and obesity). Age and DM (p<0.1 and >0.05) associated with global prevalence of high risk for OSAS (Table 3).

**Table 2:** Scores of the Berlin Questionnaire (BQ) responses and prevalence of high risk for OSAS per BQ categories and globally

Questions in the scoring categories	N (%)
**Do you snore?**	
Yes	403 (65.4)
**You snoring is...**	
Louder than talking or much louder than talking	138 (22.4)
**How often do you snore?**	
3-4 times per week or almost every day	136 (22.1)
**Has your snoring ever bothered other people?**	
Yes	219 (35.6)
**Has anyone noticed that you stop breathing during your sleep?**	
3-4 times per week or almost every day	11 (1.8)
**How often do you feel tired or fatigued after your sleep?**	
3-4 times per week or almost every day	96 (15.6)
**During your waking time, do you feel tired or not up to par?**	
3-4 times per week or almost every day	96 (15.6)
**Have you ever nodded off or fallen asleep while driving a vehicle?**	
Yes	21 (3.4)
**Risk for OSAS**	
Category I	306 (49.7)
Category II	63 (10.2)
Category III	478 (77.6)
Global	261 (42.4)

OSAS: obstructive sleep apnea syndrome.

**Table 3:** Prevalence of risk for OSAS^1^ defined via the Berlin Questionnaire per category and globally, according to risk variables

**Risk for OSAS**												
	**Category I**			**Category II**			**Category III**			**Global**		
	**N (%)**	**N (%)**	**p value**	**N (%)**	**N (%)**	**p value**	**N (%)**	**N (%)**	**p value**	**N (%)**	**N (%)**	p value*^(3^
	Yes	No		Yes	No		Yes	No		Yes	No	
**Sex**			0.339			0.074			0.001			0.308
Female	183 (48.0)	198 (52.0)		46 (12.1)	335 (87.9)		313 (82.2)	68 (17.8)		168 (44.1)	213 (55.9)	
Male	123 (52.3)	112 (47.7)		17 (7.2)	218 (92.8)		165 (70.2)	70 (29.8)		93 (39.6)	142 (60.4)	
**Age group**			<0.001			0.156			<0.001			<0.001
45-49	49 (44.5)	61 (55.5)		9 (8.2)	101 (91.8)		68 (61.8)	42 (38.2)		32 (29.1)	78 (70.9)	
50-59	139 (56.5)	107 (43.5)		32 (13.0)	214 (87.0)		195 (79.3)	51 (20.7)		124 (50.4)	122 (49.6)	
60-69	81 (54.0)	69 (46.0)		17 (11.3)	133 (88.7)		116 (77.3)	34 (22.7)		68 (45.3)	82 (54.7)	
70-79	33 (38.4)	53 (61.6)		4 (4.7)	82 (95.3)		77 (89.5)	9 (10.5)		33 (38.4)	53 (61.6)	
80-99	4 (16.7)	20 (83.3)		1 (4.2)	23 (95.8)		22 (91.7)	2 (8.3)		4 (16.7)	20 (83.3)	
**Diabetes**			0.412			0.516			<0.001			0.105
Yes	80 (53.0)	71 (47.0)		13 (8.6)	138 (91.4)		131 (86.8)	20 (13.2)		73 (48.3)	78 (51.7)	
No	223 (48.7)	235 (51.3)		49 (10.7)	409 (89.3)		340 (74.2)	118 (25.8)		185 (40.4)	273 (59.6)	
**Hypertension**			1.00			1.000						
Yes	223 (49.8)	225 (50.2)		43 (9.6)	405 (90.4)							
No	83 (49.4)	85 (50.6)		20 (11.9)	148 (88.1)							
**^(2^BMI**			<0.001			0.498						
BMI≥30	116 (61.4)	73 (38.6)		22 (11.6)	167 (88.4)							
BMI<30	190 (44.5)	237 (55.5)		41 (9.6)	386 (90.4)							

1 OSAS: obstructive sleep apnea syndrome;

2 BMI: body mass index.

3 Pearson chi-square test, with continuity correction for dichotomous risk variables.

Table 4 shows the difference of the means of age, SBP, DBP and BMI according to the presence of high risk for OSAS. In category I, there was association with age, BMI (p<0.01) and DBP (p<0.1 and >0.05). In category II, none of the four variables associated with high risk for OSAS. In category III, the elderly showed higher prevalence of high risk for OSAS. No statistically significant association was observed with global prevalence of high risk for OSAS. In categories III and global, SBP, DBP and BMI were not assessed.

**Table 4:** Difference of the means according to the presence of high risk for OSAS defined via the Berlin Questionnaire per category and globally

Risk for sleep apnea via Berlin Questionnaire												
	**Category I**		**p value**	**Category II**		**p value**	**Category III**		**p value**	**Global**		p value
	**Yes**	**No**		**Yes**	**No**		**Yes**	**No**		**Yes**	**No**	
	**Mean±SE**	**Mean±SE**		**Mean±SE**	**Mean±SE**		**Mean±SE**	**Mean±SE**		**Mean±SE**	**Mean±SE**	
Age	57.94±0.50	60.45±0.65	<0.002	57.90±1.02	59.36±0.44	0.201	60.18±0.48	55.85±0.75	<0.001	58.77±0.55	59.53±0.59	0.347
SBP	137.55±1.23	137.78±1.35	0.899	136.53±3.13	137.80±0.95	0.676						
DBP	83.60±0.71	81.843±0.69	0.078	82.47±1.61	82.75±0.52	0.865						
BMI	28.92±0.32	27.21±0.29	<0.001	28.91±0.77	27.97±0.23	0.200						

OSAS: obstructive sleep apnea syndrome; SBP: systolic blood pressure; SE: standard error; DBP: diastolic blood pressure; BMI: body mass index. Student t test.

Table 5 shows the results of logistic regressions including the variables with p <0.2 in bivariate analyses. After adjusting, BMI (positive association) and age (negative association) maintained a statistically significant association with high risk for OSAS in category I. Considering that only sex showed association in category II, no adjustment was necessary. In category III, sex (female), age (positive) and DM remained statistically significant at level 0.05. Regarding global prevalence of high risk for OSAS, DM lost statistical significance.

**Table 5:** Adjusted OR by logistic regression of risk for OSAS defined via the Berlin Questionnaire per category and globally

**Variables**	**Category I**	**Category III**	**Global risk category**
	**ORa (95%CI)**	** ORa (95%CI)**	** ORa (95%CI)**
Age (continuous)	0.99 (0.98-0.99)	1.00 (1.00-1.01)	
**Age group**			
45-49			1
50-59			2.3 (1.30-3.50)
60-69			1.04 (0.53-2.04)
70-79			1.70 (0.98-2.94)
80-99			0.49 (0.10-2.41)
Sex (Female)		1.17 (1.06-1.29)	
Diabetes		0.87 (0.81-0.95)	1.33(0.88-2.00)
PAD continuous	1.00 (0.98-1.00)		
BMI≥30	1.02 (1.01-1.03)		

OSAS: obstructive sleep apnea syndrome; DBP: diastolic blood pressure; BMI: body mass index; ORa: adjusted OR.

## Discussion

In the present study, the BQ use showed a 42.4% global prevalence of high risk for OSAS, slightly higher than that found in two studies conducted in the city of São Paulo. In the first study, Tufik et al.,^4^ assessing the general population, have reported a 32.8% prevalence. The second study, assessing railroad workers, has reported a 35.03% prevalence.^7^ The diagnosis of OSAS in both studies was based on polysomnography. The prevalence of OSAS in different scenarios varies according to the distribution of sex, age groups, socioeconomic levels and obesity in the population.^4,8^ Lemos et al.,^9^ assessing truck drivers in São Paulo, have reported an 11.5% prevalence of high risk for OSAS, estimated using the BQ. Their study involved young and slim patients, mostly men. Another study conducted in 40 primary care units, 8 in Germany, 6 in Spain and 26 in the United States, using the BQ, has reported prevalences of high risk for OSAS varying from 19.9% in Springfield, USA, to 66.7% in Louisville, USA.^6^

The calculation of global high risk for OSAS via BQ combines the risks of three categories, and all hypertensive and/or obese individuals are classified as at risk in category III. Obesity has been strongly associated with OSAS. Tufik et al.^4^ have found an OR of 10.5 (95%CI: 7.1-15.7). Association with SAH seems to be less intense, even considering patients whose blood pressure does not drop during sleep (non-dipper), or those with resistant hypertension [odds ratio (OR) of 2.27 (95%CI: 1.76-2.92),^10^ 4.4 (95%CI: 1.2-16.31)^11^ and 7.74 (95%CI: 2.43-24.64),^12^ respectively]. The authors of the BQ do not justify the inclusion of that category and have not measured its impact on the calculation of high risk for OSAS.^3^

In our study population, 72.7% of the individuals were classified as hypertensive, and 30.7%, as obese, increasing the prevalence of high risk for OSAS in category III, and, consequently, of global prevalence. In the study by Tufik et al.,^4^ with OSAS prevalence slightly lower than that of high risk for OSAS found in this study, mean age was smaller, as was the prevalence of obesity (21.5%). Those authors have provided no data on blood pressure. A North American study,^13^ in which mean age and prevalence of obesity (25%) and of SAH (29%) were lower than those found in our study, has reported a 27% prevalence of high risk for OSAS. High prevalence of risk for OSAS in category III has also been reported by Netzer et al.^6^ in Stuart, Florida (68.8%), closer to the prevalence in category III found in the present study (77.6%).

Considering that, an overestimated prevalence of SAH and obesity could be suspected, and consequently, of OSAS. According to the 2011 and 2012 Brazilian surveillance system of risk factors and protection against chronic diseases via telephone Vigitel (2011 and 2012), the prevalence of self-reported diagnosis of hypertension in the city of Rio de Janeiro was 59.7%, the highest among all Brazilian capitals and the highest mean prevalence of all cities investigated for the age group ≥65 years. These figures are smaller than the 77.6% found in this study for the population cared for by the Niterói FDP. Regarding obesity, the Brazilian prevalence for the age groups of 45 years and older was higher (20% *versus* 30.7%). It is worth noting, however, that the prevalence of obesity recorded in Vigitel (2011 and 2012) was higher among women and less educated individuals, major groups in the present study.^14,15^

Primary snoring is believed to be the first stage of severe OSAS, and its intensity is known to associate with the severity of OSAS.^16^ Snoring has 82.6% sensitivity and 43% specificity to diagnose OSAS,^17^ thus the need to be associated with other elements to define high risk for that syndrome. In our study, the frequency of snoring was higher (65.4%) than in two other studies using the BQ (52.2% and 59%).^3,13^ Considering the higher percentage of obese individuals in our study, that discrepancy was expected. Our prevalences were lower than those of the two studies, differing in the responses to the questions *How often do you snore?, Has your snoring ever bothered other people?* and mainly *Has anyone noticed that you stop breathing during your sleep?*. However, the prevalence of high risk for OSAS in category I (49.7%) was similar to those estimated in most North American and European primary care clinics assessed by Netzer et al.^6^

Non-restoring sleep and fatigue are common in adults with OSAS.^17^ The frequencies of those conditions vary in different populations. In the article by Netzer et al.,^6^ the only question relates to dozing off or sleeping while driving, and the responses varied from 4% to 32%; in our study, we observed 3.4%, similar to the smallest value reported by Netzer et al.^6^ In the population cared for by the FDP, few individuals drive a motor vehicle. However, in places where women predominate, a lower prevalence of risk for OSAS in category II is expected, because they complain less than men do.^18^

The comparison of the prevalence of high risk for OSAS in the United States and Europe shows similar results in category I (43.1% and 43.5%). However, the prevalence of high risk for OSAS in category II (daytime sleepiness/fatigue) differed in those areas, being three-times higher in the United States than in Europe (32.4% and 11.8%).^6^ In our study, the prevalence of high risk for OSAS in category II was closer to the European one (10.2%).

Hypertension has been associated with OSAS, in studies both using polysomnography^19^ and estimating the high risk for OSAS via questionnaires, regardless of other risk factors.^20^ The OR found in such studies were greater than 2. In a critical review, Mohsenin^21^ states that daytime hypertension is present in up to 60% of patients with OSAS. In the present study, only the difference of mean DBP associated with high risk for OSAS in category I, and such association disappeared (ORa=1) after adjusting for age and BMI. Margallo et al.^22^ have estimated the association of blood pressure changes with high risk for OSAS according to the modified BQ, with risk exclusion in category III. Their results are comparable to those observed in our study with statistically significant difference only for mean DBP.

The interruptions in airflow lead to brief awakenings that cause daytime sleepiness and fatigue.^17^ The BQ is aimed at capturing those changes by using questions grouped into categories I and II. However, the frequency of positive responses to those questions varies culturally, as observed from the prevalence differences between North American and European communities.^6^

In our study, the BQ reliability, tested with Cronbach alpha, was 0.586 (weak) for category I. Cronbach alpha increased to 0.618 (acceptable) by withdrawing the question *Has anyone noticed that you stop breathing during your sleep?*. For category II, Cronbach alpha was 0.521 (weak). By withdrawing the question *Have you ever nodded off or fallen asleep while driving a vehicle?*, Cronbach alpha increased to 0.705 (acceptable). Assessing BQ validation, Cronbach alpha reached higher levels: 0.92 for category I and 0.86 for category II, when excluding the question *Have you ever nodded off or fallen asleep while driving a vehicle?*. This can be attributed to the low educational level of most individuals assessed, as well as to the higher percentage of women in the study sample, which might have yield false negative responses, mainly in category II.

The present study has some limitations worth noting. First, due to its cross-sectional nature, we could not establish whether SAH or obesity preceded the occurrence of OSAS. Second, there were 35% of losses (individuals who refused to participate in the study), mainly among men and individuals of the two extreme age groups (45 to 49 years and 80 to 99 years). The participation of a larger number of ill individuals, such as hypertensives, might have led to overestimation of the prevalence of OSAS.

## Conclusion

The global prevalence of high risk for OSAS, estimated via BQ, in the population cared for by the FDP was 42.4%. However, because of the losses, that prevalence might have been overestimated. In addition, the high frequency of SAH and obesity increased the prevalence of risk for OSAS. The prevalences in the three BQ categories were very different, but comparable to those reported in the literature. The BQ reliability was lower in this study population, whose educational level is lower than that of other populations studied. Our data show that the BQ reliability in populations mainly formed by female and low-educational-level individuals increases when excluding from the analysis the questions *Has anyone noticed that you stop breathing during your sleep?* and *Have you ever nodded off or fallen asleep while driving a vehicle?*, indicating the importance of performing new studies to validate that tool for that group.
